# AKT Signaling Differentially Regulates the Expression of Two Evolutionarily Conserved *Wnt5a* Isoforms in Lung Mesenchymal Cells

**DOI:** 10.3390/cells15090843

**Published:** 2026-05-04

**Authors:** Susan M. Smith, Jing C. Zhou, Hongqiao Zhang, Rutuja Kibe, Jason Chwa, Zhaoxia Qu, Beiyun Zhou, Parviz Minoo, Changgong Li

**Affiliations:** 1 Division of Neonatology, Departments of Pediatrics, LAC + USC Medical Center and Children’s Hospital, Los Angeles, CA 90033, USA; 2 Hastings Center for Pulmonary Research, Keck School of Medicine, University of Southern California, Los Angeles, CA 90033, USA; 3 Department of Immunology and Immune Therapeutics, Keck School of Medicine, University of Southern California, Los Angeles, CA 90033, USA; 4 Norris Comprehensive Cancer Center, Keck School of Medicine, University of Southern California, Los Angeles, CA 90033, USA; 5 Kapiolani Medical Center for Women and Children, University of Hawaii, Honolulu, HI 96826, USA; 6 Division of Pulmonary, Critical Care and Sleep Medicine, Department of Medicine, Keck School of Medicine, University of Southern California, Los Angeles, CA 90033, USA

**Keywords:** WNT5a isoforms, AKT, ERK, WNT signaling, fibrosis, lung injury

## Abstract

WNT5a is a lipid-modified glycoprotein member of the WNT family of signaling molecules. Two isoforms of WNT5a have been identified that are conserved across mice and humans. These isoforms display specific functions in regulating cancer cell activities. While WNT5a is, indeed, essential for normal lung development and homeostasis, and is dysregulated in multiple lung diseases, little to no information is available regarding the expression or potential function of WNT5a isoforms in normal or diseased lungs. Such information has the potential to help to elucidate the more precise and nuanced functions of WNT5a in various pulmonary conditions. In this study, we characterized the expression of individual *Wnt5a* isoforms during mouse lung development and compared their expression across major alveolar cell populations. We further investigated the molecular basis of the signaling mechanisms that regulate *Wnt5a* isoform expression in fibroblasts, the major lung cell type with high-level *Wnt5a* expression. We present data that reveal a role for the AKT pathway in differentially regulating the expression of *Wnt5a* isoforms, a novel finding. Furthermore, we demonstrate that *Wnt5a* isoforms are dysregulated in bleomycin-induced fibrosis and Pseudomonas aeruginosa (PA)-induced acute lung injury and exhibit distinct impacts in *Wnt5a* isoform expression in response to lung injury.

## 1. Introduction

WNT5a is a lipid-modified glycoprotein belonging to the WNT family of signaling molecules, which mainly signals through the β-catenin (CTNNB1)-independent noncanonical WNT pathways via the ROR and Frizzled (FZD) family of receptors but can also activate canonical WNT signaling in the presence of certain receptors such as FZD4 [1,2,3,4,5]. WNT5a has been shown to play key roles in many aspects of lung biology. We and others have demonstrated that WNT5a is essential for both embryonic and neonatal lung development, as well as lung homeostasis by regulating differentiation and proliferation of alveolar myofibroblasts (AMFs, also named secondary crest myofibroblasts) and defining the state of type 1 and type 2 alveolar epithelial cells (AT1s and AT2s) in the lung [6,7,8,9,10,11]. Recent studies have demonstrated that WNT5a is dysregulated and may be involved in the pathogenesis of multiple lung diseases including bronchopulmonary dysplasia (BPD), chronic obstructive pulmonary disease (COPD), idiopathic pulmonary fibrosis (IPF), acute respiratory distress syndrome (ARDS), and pulmonary artery hypertension [8,12,13,14,15,16,17,18]. The detailed mechanisms that mediate the expression and functions of WNT5a remain to be further investigated.

Two isoforms of WNT5a, WNT5a-Long (WNT5a-L), and WNT5a-Short (WNT5a-S), are conserved across mice and humans [19,20]. The WNT5a-S protein is truncated at the N-terminal by 15 (in human) or 20 (in mouse) amino acids compared to WNT5a-L; their expression is controlled by distinct promoters [19]. Differential expression of *Wnt5a* isoforms has been reported in both normal tissues and in tumor cells [20,21,22,23,24]. Importantly, the two WNT5a isoforms exhibit distinct functions in regulating cell proliferation. While WNT5a-L inhibits, WNT5a-S activates proliferation; elevated WNT5a-S contributes to the increased proliferation of cancer cells [20]. These data support that the differential expression and function of WNT5a isoforms may underlie its complex roles in many biological and pathological processes. However, the difference of WNT5a isoforms has not been fully appreciated in the interpretation of WNT5a’s role in lung diseases.

*Wnt5a* expression is regulated by several signaling pathways and transcriptional factors. Both TGF-β signaling and FOXF1 transcriptional factor activate *Wnt5a* expression in lung fibroblasts [15,25]. In airway smooth muscle cells, TGF-β activates *Wnt5a* expression to induce extracellular matrix production [26]. Whether these regulatory mechanisms are isoform-specific remains unknown. *Wnt5a* expression also requires NF-kB signaling in neonatal lung fibroblast [18]. A previous study reported that both TNF-alpha and NF-kB predominantly regulate *Wnt5a-S* expression in NIH-3T3 cells [19]. However, it remains unclear whether these regulatory mechanisms are isoform-specific in the lungs.

In the current study, we characterized the expression of *Wnt5a* isoforms during normal lung development, homeostasis, and in major alveolar cell types of neonatal lungs. Our data show that *Wnt5a* isoforms are highly expressed in fibroblasts expressing Platelet-Derived Growth Factor Receptor alpha (*Pdgfra*^+^) and that *Wnt5a-S* is differentially expressed between fibroblast subpopulations distinguished by *Pdgfra* levels. To explore the underlying mechanisms, we investigated the roles of Platelet-Derived Growth Factor-AA (PDGF-AA) and its downstream intracellular mediators in regulating *Wnt5a* isoform expression and discovered a novel mechanism involving AKT serine/threonine kinase-mediated differential regulation of *Wnt5a* isoforms in lung fibroblasts. During lung fibrosis, both *Wnt5a* isoforms are increased. Further analysis revealed that the Transforming Growth Factor beta (TGF-β), the key mediator of lung fibrosis, activates *Wnt5a* isoform expression through the activities of SMAD, ERK and AKT pathways. Furthermore, we assessed dysregulation of *Wnt5a* isoforms in Pseudomonas aeruginosa (PA)-induced acute lung injury and found selective inhibition of *Wnt5a-L* during PA-induced ALI, demonstrating a distinct *Wnt5a* response in different disease models.

## 2. Materials and Methods

### 2.1. Mouse Breeding

Mice were purchased from the Jackson laboratory (Bar Harbor, ME, USA) and housed in pathogen-free conditions with a 12-h light/dark cycle in the animal facility of University of Southern California. The animal housing facilities are fully accredited by the American Association for Accreditation of Laboratory Animal Cares (AAALAC). All animal studies were performed according to protocols approved by the USC Institutional Animal Care and Use Committee (IACUC). *Aqp5cre;mTmG* mice were generated by breeding the *Aqp5cre* mice [27] with the *mTmG* mice (JAX007676). For injury studies, mice were randomly assigned to each control or treatment group; the lungs were treated and analyzed in random sequence. Bleomycin injury was conducted as described previously [28]. Briefly, C57BL6 adult mice (2 to 3 months of age) were anesthetized with ketamine and xylazine followed by intratracheal administration of bleomycin (one dose, 0.75 U/kg) or sterile PBS. Mice were sacrificed for lung histology analysis and RNA extraction on day 7, 14, and 21 after bleomycin administration. For PA treatment, FVB/N mice (2 to 3 months of age) were infected with 2 × 10^7^ CFU/mouse pseudomonas aeruginosa or PBS via intratracheal instillation [29]. Mice were euthanized 24 h post infection. Mice were monitored daily and euthanized if there was an unexpected illness. All control and injured lungs were collected by an investigator who was blind to the group allocation and analyzed for histology or biochemistry assays.

### 2.2. Isolation, Culture, and Treatment of Lung Fibroblasts

Lung fibroblasts were isolated from postnatal day 5 (P5) pups or 2–3-month adult mice, as described [8]. Briefly, lungs were dissected in Hank’s balanced salt solution (HBSS), inflated, and digested with Dispase at 37 °C for 15 min. After removing major airways, lung tissues were dissociated with a gentle MACS dissociator (Miltenyi Biotec. Inc., San Diego, CA, USA) and filtered with 40 μm cell strainers. The dissociated cells were collected by centrifugation, resuspended in DMEM containing 10% FBS, and plated in cell-culture plates at 37 °C with 5% CO_2_ for 1 h. After removing the floating cells, the attached fibroblasts were washed with PBS and re-cultured in a fresh medium a few times within the first 20 h. Cells grown to near confluence were trypsinized and stored in a storage medium containing 10% FBS and 10% DMSO in DMEM in liquid nitrogen for future use. Cells lower than passage 5 were plated in 12-well plates in DMEM with 10% FBS until near confluence, starved in an FBS-free medium, and treated with growth factors, inhibitors or bleomycin, as specified in each experiment. For PA treatment, cells grown to near confluence were changed to an antibiotic-free medium, then infected with a 2 × 10^6^ CFU PA/well or vehicle. Six hours after infection, cells were collected for RNA extraction.

### 2.3. RNA Isolation and Real-Time Quantitative Polymerase Chain Reaction (qRT-PCR)

RNA was isolated from cultured cells with Trizol reagents (ThermoFisher Scientific, San Jose, CA, USA) and Zymo Direct-zol RNA microprep kit (Zymo Research, Irvine, CA, USA). DNA contamination was removed by DNase I supplied with the kit. RNA quality was determined by the ratio of OD260/OD280. One microgram of RNA was used in each reaction to synthesize cDNA with the EasyScript Plus cDNA synthesis kit following the manufacturer’s protocol (Lamda Biotech, Inc., Ballwin, MO, USA).

Quantification of selected gene expression by qRT-PCR was conducted using QuantStudio 7 Pro Real-Time PCR System (ThermoFisher Scientific, San Jose, CA, USA) and PowerUp SYBR Green Master Mix. The expression of *Wnt5a* isoforms was analyzed using TaqMan Fast Advanced Master Mix (ThermoFisher Scientific, San Jose, CA, USA) with primer/probe sets, as previously published [19]. Expression levels of target genes were normalized with that of TBP (TATA-box binding protein) and compared between samples using the ΔΔCt method [30]. Sequences of primers and probes are listed in Supplementary Table S1.

### 2.4. Western Blot Analysis

Protein extracts were prepared from the cultured cells using radioimmunoprecipitation assay buffer (RIPA buffer, Sigma, St. Louis, MO, USA) and separated on 4–12% Tris–Glycine gels (ThermoFisher Scientific, San Jose, CA, USA). Proteins were blotted onto PVDF membranes, probed with antibodies, and analyzed with the SuperSignal West Femto Maximum Sensitivity Substrate, as described by the manufacturer (ThermoFisher Scientific, San Jose, CA, USA). The primary antibodies used for Western blot are listed in Supplementary Table S2.

### 2.5. Immunostaining

Neonatal and adult mouse lungs were fixed in 4% formaldehyde, dehydrated, and embedded in paraffin. Five micrometer tissue sections were prepared and used for hematoxylin and eosin (H and E) staining and immunofluorescence staining, as previously described [31]. Immunofluorescence-stained sections were counterstained with 4′,6′-diamidino-2-phenylindole (DAPI). The primary antibodies used for immunofluorescence staining are listed in Supplementary Table S2. For collagen staining, paraffin sections of the control and bleomycin-injured lungs were rehydrated through ethanol gradient and then stained with Picrosirius Red (0.1%)/Fast Green FCF (0.1%) solutions for 1 h, followed by 0.01 N HCl for 2 min. Sections were dehydrated through ethanol solutions and mounted with Permount.

### 2.6. Single-Cell RNA-Sequencing (scRNA-Seq) Data Analysis

Single-cell RNA-seq data from mouse postnatal day 7 (P7) lung were obtained from Hurskainen et al. [32]. Demultiplexed sequencing datasets processed with CellRanger v3.0.2—GSM4594484 (Lung_P3_P7_1), GSM4594485 (Lung_P3_P7_2), GSM4594486 (Lung_P3_2), GSM4594487 (Lung_P7_1), GSM4594488 (Lung_P14_1), and GSM4594489 (Lung_P14_2)—were downloaded from the NCBI Gene Expression Omnibus (GEO) under accession GSE151974 and imported into Partek Flow (v12.6.0) for downstream analysis.

All six datasets were normalized jointly using counts per million (CPM) plus log2(x + 1). Cells from the P7 sample (Lung_P7_1) were then subset, yielding 3664 cells from hyperoxia-treated mice and 4337 cells from normoxia-treated controls. Principal component analysis (PCA) was performed using the top 2000 most variable genes. Graph-based clustering was conducted using the Louvain algorithm (30 nearest neighbors, 15 principal components, 10 iterations, resolution = 0.5). UMAP dimensionality reduction was computed using 10 principal components, a neighborhood size of 30, and a minimum distance of 0.3.

Major cell types were identified based on canonical marker gene expression: epithelial (*Nkx2.1*), endothelial (*Cdh5*), mesenchymal (*Col1a1*), and immune (*Ptprc*). Mesenchymal cells were subset and reanalyzed using PCA, Louvain clustering (same parameters as above), and UMAP. This analysis identified five mesenchymal subclusters in the lungs under normoxia.

Differential expression between mesenchymal clusters 2 and 1 under normoxia condition was assessed using ANOVA in Partek Flow. A total of 715 genes (fold change > 2, *p* < 0.00005) were identified and subsequently analyzed using Ingenuity Pathway Analysis (QIAGEN, Germantown, MD, USA) for signaling pathway and cell function analysis.

### 2.7. Statistical Analysis

Statistical analyses were performed with GraphPad Prism (version 9.5.1). Quantitative data are presented as mean values ± standard error of the mean (SEM). *p*-values were calculated by 2-tailed Student’s *t* tests and Wilcoxon tests, with *p* < 0.05 considered statistically significant (indicated by asterisk); *n* represents independent biological repeats in each experiment. Sample sizes were based on prior publications and ethical considerations for animal welfare.

## 3. Results

### 3.1. Both Wnt5a Isoforms Peak at P7

**Figure 1:**
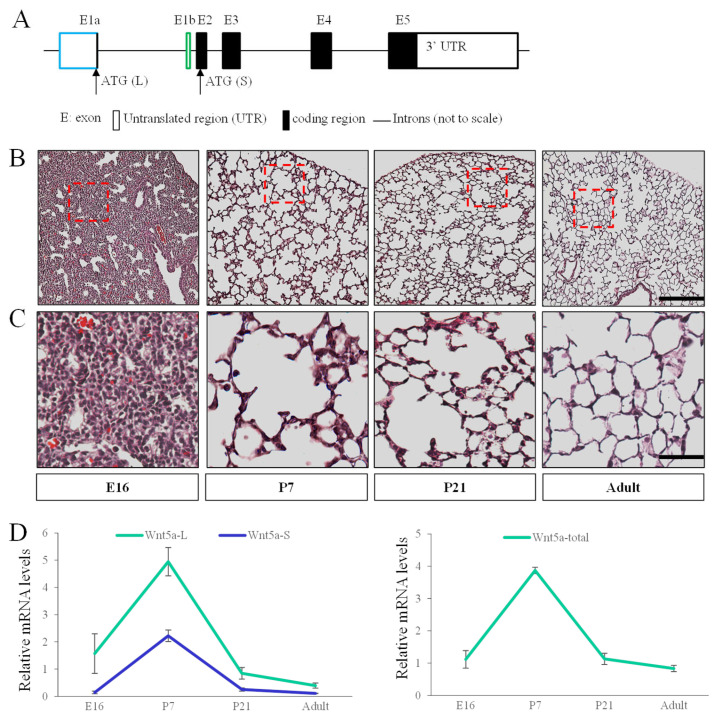
Developmental expression pattern of *Wnt5a* isoforms: (**A**) illustration of mouse *Wnt5a* gene. The boxes indicate exons (E) and the lines indicate introns (not to scale). Transcription of *Wnt5a-Long* (*Wnt5a-L*) and *Wnt5a-Short* (*Wnt5a-S*) is controlled by distinct promoters. *Wnt5a-L* transcript contains E1a-E2-E3-E4-E5 and *Wnt5a-S* transcript contains E1b-E2-E3-E4-E5. Upright arrows indicate translation start sites of *Wnt5a-L* and *Wnt5a-S* located in E1a and E2, respectively. Exon with blue border (E1a) is unique for *Wnt5a-L* and exon with green border (E1b) is specific for *Wnt5a-S*. Exons with black borders are used by both isoforms; (**B**,**C**) H and E staining shows mouse lung morphology at embryonic day 16 (E16), postnatal day 7 (P7), P21 and adult. Panel (**C**) shows higher magnification of boxed areas in panel (**B**) of each stage. Scale bar: 200 μm in (**B**), 50 μm in (**C**); and (**D**) qRT-PCR shows expression pattern of *Wnt5a* isoforms and total *Wnt5a* during lung development and homeostasis. Data indicate mean ± SEM. n = 3 for each time point.

WNT5a proteins have two major isoforms encoded by two mRNA variants in mice (Figure 1) [19,20]. Using isoform specific primers [19], we characterized the expression pattern of *Wnt5a-S* and *Wnt5a-L* during lung development from E16, P7, P21 to adult (2 months) by qRT-PCR analysis (Figure 1). Our data show that *Wnt5a-L* expresses at a higher level than that of *Wnt5a-S* in the lung from E16 to adult (Figure 1). Expression of both isoforms peak around postnatal day 7 (P7), the stage of alveologenesis. Similar expression patterns were observed in total *Wnt5a* levels (Figure 1).

### 3.2. Wnt5a-S and Wnt5a-L Are Predominantly Expressed in Pdgfra^+^ Cells with Higher Wnt5a-S/Wnt5a-L Ratio in Pdgfra-H Subpopulation at P7

**Figure 2:**
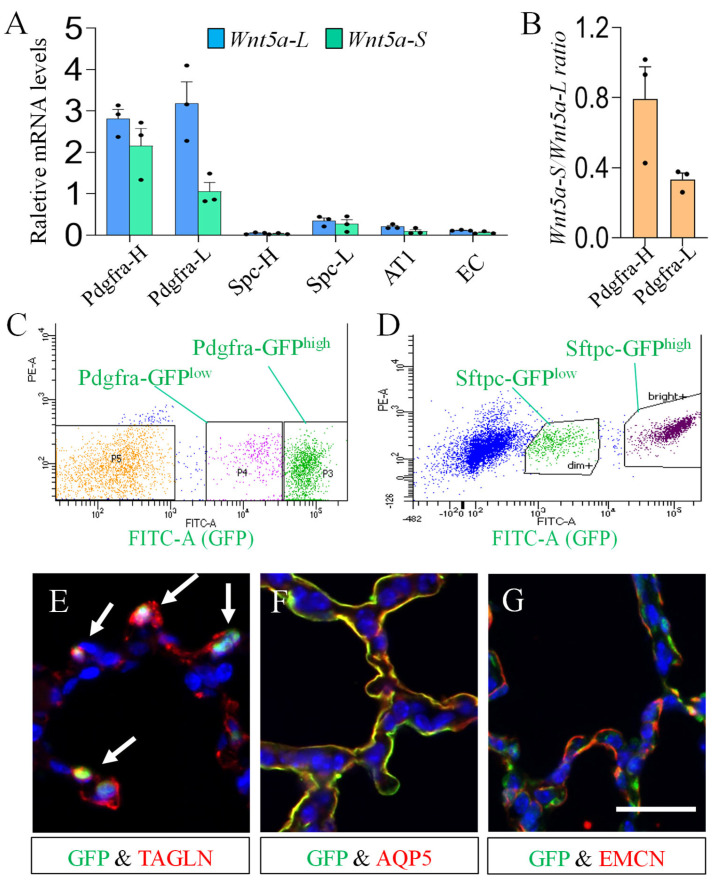
Cell-type specific expression of *Wnt5a-L* and *Wnt5a-S*: (**A**) relative mRNA levels of *Wnt5a-L* and *Wnt5a-S* (normalized with *TBP* by qRT-PCR) in *Pdgfra*-GFP^high^ (*Pdgfra*-H) and *Pdgfra*-GFP^low^ (*Pdgfra*-L) mesenchymal cells, *Sftpc*-GFP^high^ (*Spc*-H) and *Sftpc*-GFP^low^ (*Spc*-L) AT2s, AT1s and endothelial cells (EC). Please note that *Wnt5a-S* transcript level is significantly higher in *Pdgfra*-H as compared to *Pdgfra*-L mesenchymal cells. Data indicate mean ± SEM. n = 3; (**B**) ratios of *Wnt5a-S* to *Wnt5a-L* in *Pdgfra*-H and *Pdgfra*-L cells; (**C**,**D**) gating strategy for FACS of *Pdgfra*-GFP^high^ and *Pdgfra*-GFP^low^ subpopulations (**C**) and *Sftpc*-GFP^high^ and *Sftpc*-GFP^low^ subpopulations (**D**); and (**E**–**G**) immunofluorescence staining shows the overlapping of GFP (green, arrows) and TAGLN (red) in *Pdgfra-EGFP* lungs (**E**), GFP (green) and AQP5 (red) in *Aqp5cre;mTmG* lungs (**F**), and GFP (green) and EMCN (red) in *Pecam1-EGFP* lungs (**G**). Scale bar: 25 μm.

Next, we determined the cell type specific expression of *Wnt5a* isoforms. *Pdgfra* is widely expressed in neonatal lung interstitial fibroblasts [33,34,35,36,37]. In P7 *Pdgfra-EGFP* (JAX007669) [38] lungs, two populations of *Pdgfra*^+^ cells depending on the *Pdgfra*-GFP levels have been identified as *Pdgfra*-GFP^high^ (*Pdgfra*-H) and *Pdgfra*-GFP^low^ (*Pdgfra*-L) cells [36,39,40]. Using the *Sftpc*-*GFP* model, we have shown that P7 lungs contain two AT2 populations depending on *Sftpc*-GFP levels, named *Sftpc*-GFP^high^ (*Spc*-H) and *Sftpc*-GFP^low^ (*Spc*-L) AT2s [9,41]. By FACS, we collected the *Pdgfra*-H and *Pdgfra*-L fibroblasts and *Spc*-H and *Spc*-L AT2s from the P7 *Pdgfra-EGFP* and *Sftpc-GFP* lungs, respectively (Figure 2). The AT1s and endothelial cells (ECs) were collected by FACS from P7 *Aqp5cre;mTmG* [27] and *Pecam1-EGFP* (JAX033111) [42] lungs, respectively.

qRT-PCR analysis revealed that both *Wnt5a-S* and *Wnt5a-L* are predominantly expressed in *Pdgfra*^+^ cells as compared to other cell types. Interestingly, levels of *Wnt5a-S* are significantly different between *Pdgfra*-H and *Pdgfra*-L fibroblasts (Figure 2), indicating differential regulation of *Wnt5a-S* in the distinct fibroblast population [39,40].

### 3.3. Differential Regulation of Wnt5a Isoform Expression by AKT Signaling in Neonatal Lung Fibroblasts

**Figure 3:**
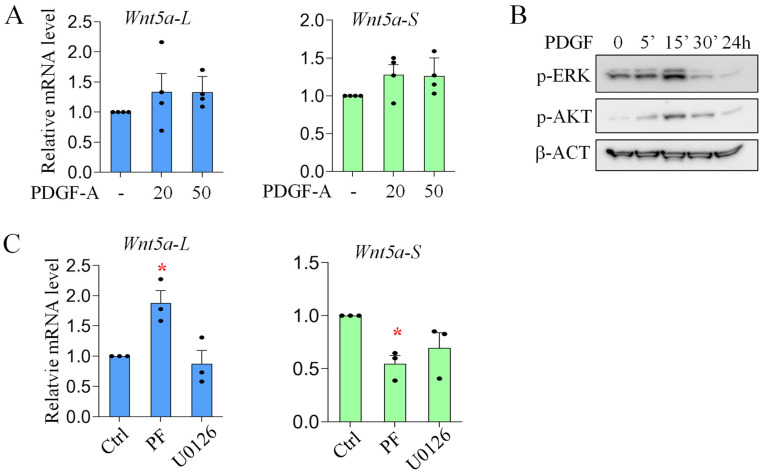
Effects of PDGF-AA on *Wnt5a* isoform expression. P5 lung mesenchymal cells were cultured and treated with (20 or 50 ng/mL) or without (-) PDGF-AA for 24 h. Levels of *Wnt5a-S* and *Wnt5a-L* were measured by qRT-PCR: (**A**) *Wnt5a-L* and *Wnt5a-S* expression in PDGF-AA-treated cells; (**B**) Western blot shows that PDGF-AA transiently increases the levels of both p-ERK and p-AKT. β-actin (β-ACT) as loading control; and (**C**) PI3k/AKT inhibitor (PF, 0.5 μM) significantly increases *Wnt5a-L* but decreases *Wnt5a-S* when compared to vehicle controls (Ctrl). Data indicate mean ± SEM. Significant changes (treated vs. untreated) are indicated by the asterisk (*p* < 0.05). n ≥ 3.

To determine whether PDGF-AA signaling causes the selective activation of *Wnt5a-S* in *Pdgfra*-H versus *Pdgfra*-L subpopulations, we examined the effects of PDGF-AA on *Wnt5a* isoform expression in primary lung fibroblasts that contain mainly *Pdgfra*^+^ cells [8]. Levels of *Wnt5a-S* and *Wnt5a-L* were measured by qRT-PCR. As shown in Figure 3, PDGF-AA did not significantly alter the expression of *Wnt5a-S* and *Wnt5a-L*. Since PDGF signaling mainly activates AKT and ERK signaling, we assessed the ATK and ERK activation in PDGF-AA treated cells using Western blot. Our data show that although both p-ERK and p-AKT levels were transiently increased by PDGF-AA, no significant increase was observed 24 h after the treatment (Figure 3). Then, we blocked AKT and ERK pathways separately in presence of PDGF-AA. As shown in Figure 3, AKT inhibitor, PF04691502, reduced *Wnt5a-S* but increased *Wnt5a-L*, indicating that AKT signaling differentially regulates *Wnt5a* isoform expression in lung fibroblasts.

**Figure 4:**
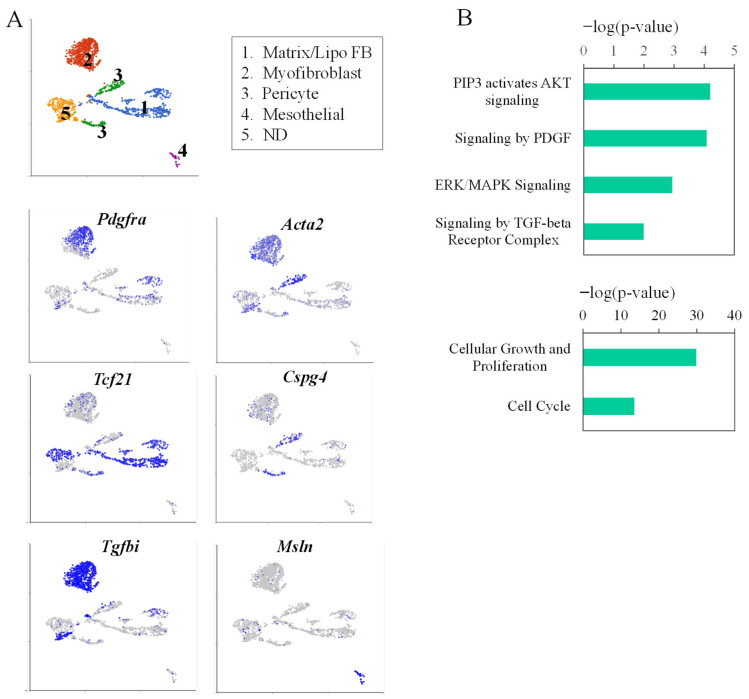
scRNA-seq analysis of P7 mesenchymal cells: (**A**) UMAP and distribution of *Pdgfra*, *Acta2* (for myofibroblast), *Tcf21* (for lipofibroblast), *Cspg4* (for pericyte), *Tgfbi* (for TGF-β signaling activity), and *Msln* (for mesothelial cells) in mesenchymal cell clusters of P7 lungs. Lipo FB: lipofibroblast; ND: non-distinguishable; and (**B**) increased PDGF, AKT, ERK and TGF-β signaling and cell proliferation in Cluster 2 as compared to that of Cluster 1 cells. Differentially expressed genes in Cluster 2 (569 cells) versus Cluster 1 (404 cells) were identified by ANOVA. A total of 715 genes identified by a fold change larger than two and a *p*-value less than 0.00005 were fed into Ingenuity Pathway Analysis (IPA) for signaling pathway analysis.

Previous studies demonstrated that the *Pdgfra*-H and *Pdgfra*-L subpopulations contain mainly alveolar myofibroblast and lipofibroblast/matrix fibroblasts, respectively, in neonatal lungs [36,39,40]. To confirm this, we analyzed scRNA-seq data of P7 mouse lung mesenchymal cells (GSE151974) [32]. Five clusters of mesenchymal cells were identified based on their gene expression profiles (Figure 4). Cluster identities were validated using gene set enrichment analysis in Enrichr “https://maayanlab.cloud/Enrichr/#metadata (accessed on 14 January 2026)” with the top 10 differentially expressed genes per cluster. As shown in Figure 4, *Pdgfra* is mainly expressed in Clusters 1 and 2, with the highest expression level in Cluster 2, which are *Acta2*-positive, representing AMFs. In contrast, Cluster 1 expresses lower levels of *Pdgfra* and is enriched with *Tcf21*, a marker for lipofibroblasts. This confirms that the *Pdgfra*-H and *Pdgfra*-L cells represent myofibroblast (Clusters 2) and lipofibroblast/matrix fibroblast (Cluster 1), respectively.

Further comparison between Clusters 2 and 1 revealed that PDGF, ATK, and ERK signaling are all elevated in Cluster 2 AMFs (Figure 4). Therefore, higher AKT activity may contribute to the higher levels of *Wnt5a*-*S* in *Pdgfra*-H cells. It should be noted that overall AKT activity is regulated by multiple signaling pathways, including PDGF and TGF-β, both of which are increased in the *Pdgfra*-H/Cluster2 AMFs (Figure 4). Since both AKT signaling and WNT5a-S promote cell proliferation [20,43], the higher levels of AKT signaling and increased *Wnt5a*-S may contribute to higher levels of cell proliferation in the *Pdgfra*-H/Cluster2 AMFs (Figure 4).

### 3.4. Increased Wnt5a Isoforms in Bleomycin-Induced Lung Fibrosis

**Figure 5:**
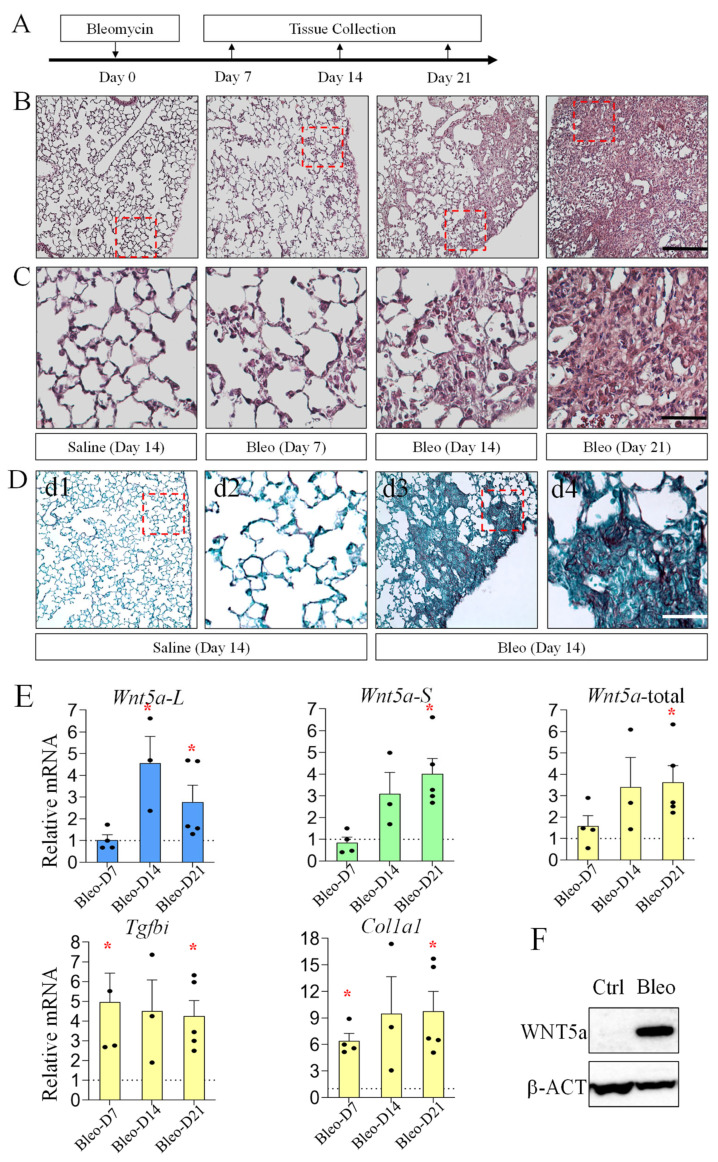
*Wnt5a* isoforms are increased in fibrotic phase of bleomycin (Bleo)-induced lung fibrosis: (**A**) illustration of experimental design; (**B**,**C**) H and E staining shows the morphology of control (PBS, day 14) and Bleo-injured lungs at day 7, day 14, and day 21. Accumulation of fibrotic tissue was observed in day 14 and day 21 Bleo-injured lungs. Panel (**C**) shows higher magnification of the boxed areas in panel (**B**) under each condition; (**D**) Picrosirius Red/Fast Green FCF staining of the control and Bleo-injured (day 14) lungs. Panels (**d2**,**d4**) show higher magnification of boxed areas in (**d1**,**d3**), respectively. Scale bar: 200 μm in (**B**,**d1**,**d3**); 50 μm in (**C**,**d2**,**d4**); (**E**) relative mRNA levels (Bleo-injured vs. PBS control) of *Wnt5a-L*, *Wnt5a-S*, total *Wnt5a*, *Col1a1*, and *Tgfbi* at day 7 (D7, Bleo n = 4, PBS n = 3), D14 (Bleo n = 3, PBS n = 2), and D21 (Bleo n = 5, PBS n = 3), as determined by qRT-PCR. Data indicate mean ± SEM. Significant changes (Bleo-injured vs. PBS control) are indicated by the asterisk (*p* < 0.05); and (**F**) Western blot of WNT5a and β-actin (β-ACT, loading control) in control and Bleo-injured lungs (D21). Data represent three independent biological repeats.

Previous studies have demonstrated that WNT5a levels were increased in fibrotic lungs [15]. To determine which isoform of *Wnt5a* is altered during the development of pulmonary fibrosis, we assessed levels of *Wnt5a-L* and *Wnt5a-S* in a mouse model of pulmonary fibrosis induced by bleomycin injury. As shown in Figure 5, bleomycin-injured lungs progressively developed fibrosis from 7 to 21 days after injury and collagen was accumulated in the fibrotic areas. Expression of both *Wnt5a* isoforms increased between day 14 and day 21 after bleomycin injury, in association with the development of fibrosis and activation of ECM expression (e.g., *Col1a1*). Consistently, WNT5a protein levels were also increased in the fibrotic lungs, as shown by Western blot analysis.

**Figure 6:**
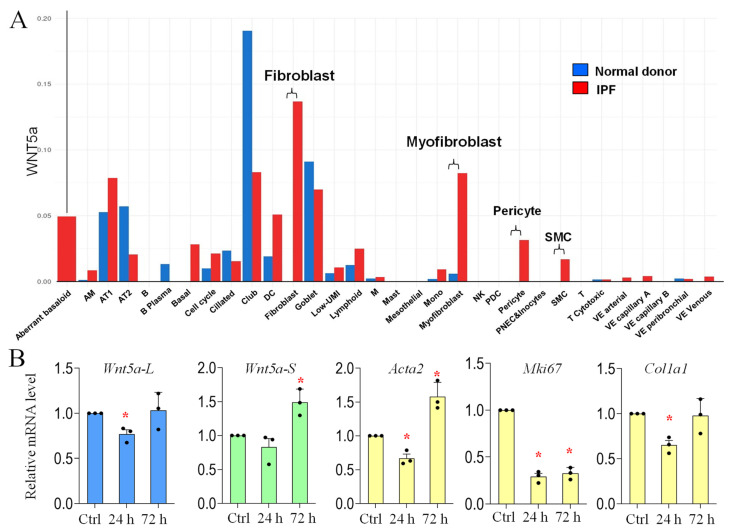
Regulation of *Wnt5a* isoform expression by bleomycin in lung fibroblasts: (**A**) *WNT5a* is increased in fibroblasts, myofibroblasts, pericytes, and smooth muscle cells (SMCs) of IPF lungs. Figure generated in IPF Cell Atlas “http://www.ipfcellatlas.com/ (accessed on 3 September 2023)” based on data by Lafyatis’s group [44]. Y-axis: *WNT5a* level. Blue bar: control lungs. Red bar: IPF lungs; and (**B**) relative mRNA levels of *Wnt5a* isoforms, *Acta2* (for myofibroblast differentiation), *Ki67* (for proliferation), and *Col1a1* (for extracellular matrix production) in control (Ctrl) or Bleo-treated (2 mUnit for 24 or 72 h) lung fibroblasts. Data indicate mean ± SEM. Significant changes (treated vs. untreated) are indicated by the asterisk (*p* < 0.05). n = 3.

In human IPF lungs, WNT5a is mainly activated in fibroblasts (Figure 6). Therefore, we assessed the expression of *Wnt5a-L* and *Wnt5a-S* in primary mouse fibroblasts treated with bleomycin. As shown in Figure 6, bleomycin moderately reduced levels of *Wnt5a-L* within 24 h but increased levels of *Wnt5a-S* by 72 h, correlating with an increase in *Acta2*. This indicates that bleomycin may not directly increase *Wnt5a* isoform expression in lung fibroblasts and that the increased *Wnt5a* in bleomycin-injured lungs may be mediated by other factors activated during lung fibrosis.

**Figure 7:**
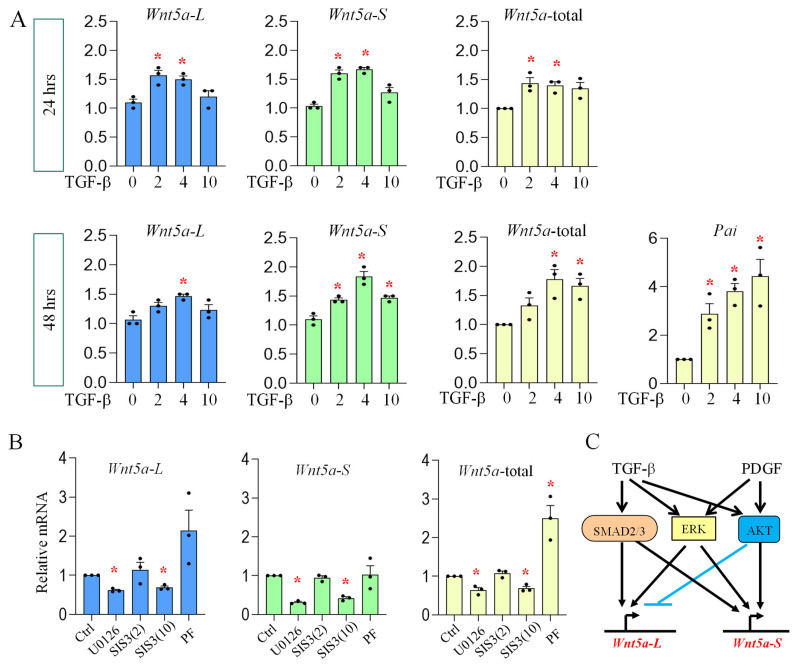
TGF-β activates expression of both *Wnt5a* isoforms: (**A**) primary lung fibroblasts were cultured and treated with (0–10 ng/mL) TGF-β1 for 24 or 48 h. Levels of *Wnt5a-L*, *Wnt5a-S*, and *total Wnt5a* were measured by qRT-PCR. *Pai* shows the effects of TGF-β1; (**B**) primary lung fibroblasts were cultured in the presence of TGF-β1 (4 ng/mL) and treated with ERK inhibitor U0126 (10 μM), SMAD inhibitor SIS3 (2 μM or 10 μM), or PI3k/AKT inhibitor PF04691502 (PF, 0.5 μM) for 48 h. Relative levels of *Wnt5a-L*, *Wnt5a-S* and total *Wnt5a* were analyzed by qRT-PCR; and (**C**) illustration of differential regulation of *Wnt5a* isoforms by PDGF and TGF-β signaling. → indicates activation, ⊣ indicates inhibition. Data indicate mean ± SEM. Significant changes (treated vs. untreated) are indicated by the asterisk (*p* < 0.05). n = 3.

It has been well-established that activation of TGF-β signaling plays a central role in lung fibrosis [45,46,47]. Consistently, *Tgfbi*, the reporter of TGF-β activity, was robustly increased in bleomycin-injured lungs (Figure 5). Thus, we investigated the effects of TGF-β on *Wnt5a* isoform expression in lung fibroblasts. As shown in Figure 7, TGF-β increased expression of both *Wnt5a* isoforms after 24 and 48 h of treatment. We further determined the role of intracellular mediators of TGF-β signaling by inhibiting SMAD, ERK, and AKT. The results show that U0126 strongly blocked, while S1S3 showed a dose-dependent inhibition on, *Wnta5-S* and *Wnt5a-L*, demonstrating that ERK and SMAD signaling positively regulate both *Wnt5a* isoforms. It should be noted that the PF04691502 increased *Wnt5a-L*, but not *Wnt5a-S*, confirming that the AKT pathway differentially regulates *Wnt5a* isoform expression (Figure 7).

### 3.5. Isoform Specific Reduction of Wnt5a-L, but Not Wnt5a-S, in PA-Induced Lung Injury

**Figure 8:**
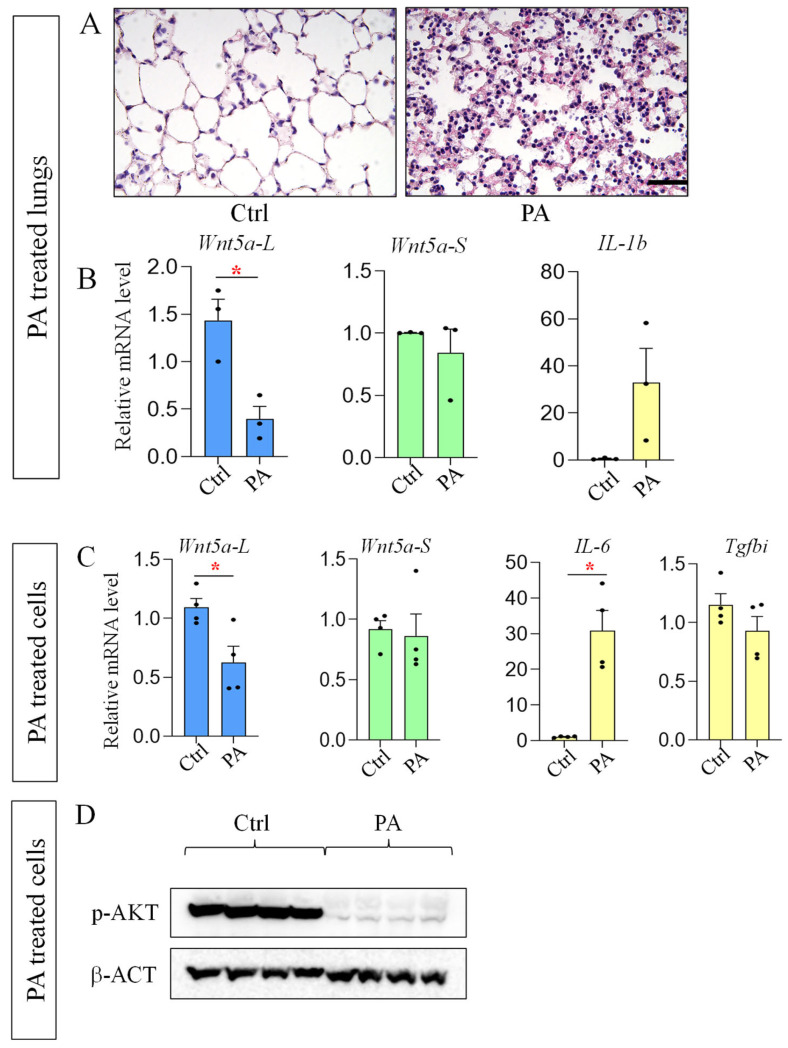
*Wnt5a-L* is selectively reduced in PA-induced ALI and PA-treated lung fibroblasts: (**A**) H and E staining shows the morphology of PBS-treated (Ctrl) and PA-injured lungs 24 h post infection. Scale bar: 50 μm; (**B**) relative mRNA levels of *Wnt5a* isoforms and *IL-1b* in PA-injured lungs (n = 3 for both Ctrl and PA-injured lungs); (**C**) relative mRNA levels of *Wnt5a* isoforms and targets of TNF-alpha (*IL-6*) and TGF-β (*Tgfbi*) signaling in control (Ctrl) or PA-treated lung fibroblasts. Data indicate mean ± SEM. Significant changes (treated vs. untreated) are indicated by the asterisk (*p* < 0.05). n ≥ 3; and (**D**) Western blot of p-AKT and β-actin (β-ACT, loading control) in control (Ctrl) or PA-treated lung fibroblasts. Each lane represents a biological repeat.

Since *Wnt5a* is also dysregulated in acute lung injury (ALI) [12,13], to further analyze the dysregulation of *Wnt5a* isoforms, we investigated their expression in PA-induced lung injury. As shown in Figure 8, the alveolar structure was disrupted after exposure to PA. Interestingly, PA selectively reduced the levels of *Wnt5a-L*, but not *Wnt5a-S*, in the injured lungs. We then isolated and treated primary lung fibroblasts with PA and found that *Wnt5a-L* is selectively repressed by PA, consistent with the in vivo observation. To determine whether the selective reduction of *Wnt5a-L* is due to increased AKT signaling, we examined levels of p-AKT by western blot. As shown in Figure 8, levels of p-AKT were robustly reduced in the PA-treated cells, indicating there might be other mechanisms that repress *Wnt5a-L* expression in PA-induced injury.

## 4. Discussion

WNT5a has been shown to play critical roles in lung development, homeostasis, and injury response. How each of the WNT5a isoforms contributes to AMF development remains to be determined. Previously, we found that overexpression of *Wnt5a-L* decreased AMFs in neonatal lungs. In the current study, we compared the expression of *Wnt5a* isoforms between *Pdgfra*-H AMFs and *Pdgfra*-L cells (containing mainly lipofibroblasts/matrix fibroblasts). As shown in Figure 2, the expression level of *Wnt5a-S*, but not *Wnt5a-L*, is selectively increased in *Pdgfra*-H AMFs. In addition, in bleomycin-treated fibroblasts, induction of myofibroblast marker *Acta2* was associated with the selective increase in *Wnt5a-S* (Figure 6). Collectively, these data imply that WNT5a-S promotes myofibroblast development.

To date, no commercial antibodies are available to distinguish between WNT5A isoforms. In addition, because WNT5A-L and WNT5A-S differ by only 15–20 amino acids, they are not readily separable by Western blot. These limit the ability to analyze WNT5a isoforms at the protein level. Due to the lack of isoform-specific reagents and models, research on the role of WNT5a isoform is hindered in research areas such as in the lung. The current study shows that both *Wnt5a* isoforms are indeed expressed, and are differentially regulated, in normal and injured lungs, supporting that potential isoform differences need to be taken into account when interpreting WNT5a functions.

One of the intriguing findings of the current study is that the AKT pathway inhibits the expression of *Wnt5a-L* but activates *Wnt5aS* (Figure 3 and Figure 7). In addition, SMAD and ERK positively regulate both isoforms in lung fibroblasts. Since both PDGF and TGF-β signaling are more activated in *Pdgfra*-H AMFs as compared to *Pdgfra*-L cells (Figure 4), the combined effects of PDGF and TGF-β signaling define the overall levels of *Wnt5a* isoforms in neonatal lung fibroblasts. For example, in *Pdgfra*-H AMFs, higher PDGF and TGF-β signaling activated SMAD, ERK and AKT pathways, which all positively regulated *Wnt5a-S* expression. On the other hand, for *Wnt5a-L*, the negative effects of AKT signaling counteracted the positive effects of SMAD and ERK, which consequently resulted in comparable levels of *Wnt5a-L* between *Pdgfra*-H AMFs and *Pdgfra*-L lipofibroblasts/matrix fibroblasts (Figure 2).

Multiple transcription factor binding sites are conserved across human and mouse *Wnt5a* promoters and intron1 [48]. These include binding sites for PPARγ, Sp1 (GC-box), C/EBP, bHLH, Smad, CUX1 (CUTL1), NF-κB, and FOX transcriptional factors. Amongst these elements, the PPARγ-, C/EBP-, and bHLH-binding sites are unique to the promoter region upstream of *Wnt5a-L*, whereas the CUX1- and NF-kB-binding sites are specific to *Wnt5a-S* promoter. In contrast, FOX binding sites are enriched within intron 1. Previous studies showed that AKT signaling regulates NF-kB and FOX family of transcriptional factors [49,50,51,52]. Whether, and how, the latter transcription factors functionally mediate the AKT-induced differential regulation of *Wnt5a* isoforms remains to be further investigated.

WNT5a has been shown to contribute to IPF pathogenesis by promoting fibroblast proliferation [15]. A previous study by Carmo-Fernandes and colleagues showed that *Wnt5a* knockout in smooth muscle cells using iSma-creERT2 protected the lung from bleomycin-induced injury, with a significantly lower deposition of collagen and a reduced number of fibrotic foci in peripheral lung tissue [53]. This study provides functional evidence on the critical role of WNT5a in the development of fibrosis. Therefore, understanding the mechanism underlying *Wnt5a* dysregulation and the functional role of each isoform in disease pathogenesis will contribute to improving treatments for pulmonary fibrosis. Although both *Wnt5a* isoforms were increased in bleomycin-injured lungs, only *Wnt5a-S* further increased during the expansion of lung fibrosis from day 14 to day 21 after bleomycin injury. This implies that WNT5a-S may play a major role in the development of pulmonary fibrosis, which remains to be functionally confirmed. To determine whether bleomycin directly increases *Wnt5a* isoform expression, we assessed the bleomycin-treated fibroblasts. Our data show that only *Wnt5a-S* was increased by bleomycin after prolonged treatment (72 h). This indicates that the increased *Wnt5a* in the fibrotic lungs may represent a secondary response to bleomycin-induced cellular injury, signaling alteration, or cell-cell communication. For example, the injured AT2s or activated immune cells may serve as the source of stimuli (e.g., TGF-β) that activates fibroblast *Wnt5a* expression in fibrotic lungs [54].

The role of WNT5a in ALI/ARDS may be context dependent. It was reported that *Wnt5a* is increased in peripheral blood mononuclear cells of SARS-CoV2 ARDS patients due to the increase in TGF-β [12]. Early activation of *Wnt5a* was also observed in septic lungs and LPS-injured fibroblasts [13]. The current study revealed that *Wnt5a-L* is selectively downregulated in PA-induced lung injury and in PA-treated lung fibroblasts. Interestingly, a recent study showed that transplantation of mesenchymal stem cells that overexpress *Wnt5a* protects the lung from LPS-induced endothelial cell injury [55]. Knowledge of the isoform of *Wnt5a* that was overexpressed in the mesenchymal stem cells and whether it directly (via secreted WNT5a) or indirectly affects the endothelial cells will help to better understand its role in this process.

## 5. Conclusions

Our results demonstrate distinct patterns of *Wnt5a* isoform expression during normal lung development and following lung injury (bleomycin-induced fibrosis and PA-induced ALI), with AKT signaling playing a critical role in their regulation. These findings suggest that each isoform may have differential roles in lung morphogenesis, as well as in the injury response and repair processes. A better understanding of the regulation and specific functions of each isoform may help to inform the development of targeted therapeutic strategies.

## Data Availability

The original contributions presented in this study are included in the article and Supplementary Materials. Further inquiries can be directed to the corresponding author.

## Supplementary Materials

The following supporting information can be downloaded at: https://www.mdpi.com/article/10.3390/cells15090843/s1, Table S1: Sequences of qRT-PCR primers. Table S2: Antibodies & Reagents.

## Abbreviations

The following abbreviations are used in this manuscript:

ALI	acute lung injury
AMF	alveolar myofibroblast
AT1	type 1 alveolar epithelial cells
AT2	type 2 alveolar epithelial cells
EGFP	Enhanced Green Fluorescent Protein
FACS	Fluorescence-Activated Cell Sorting
FBS	Fetal bovine serum
GFP	Green Fluorescent Protein
mTmG	*Gt*(*ROSA*)*26Sor^tm4^*^(*ACTB-tdTomato*,-*EGFP*)*Luo*^/*J* mice
PA	Pseudomonas aeruginosa
PBS	Phosphate-buffered saline
PDGF-AA	Platelet-Derived Growth Factor-AA
Pdgfra	platelet-derived growth factor receptor alpha
Pdgfra-H	Pdgfra-GFP^high^
Pdgfra-L	Pdgfra-GFP^low^
SEM	standard error of the mean
Spc-H	Sgtpc-GFP^high^
Spc-L	Sftpc-GFP^low^
TBP	TATA box-binding protein
TGF-β	Transforming Growth Factor beta
Wnt5a-S	Wnt5a-Short
Wnt5a-L	Wnt5a-Long
