# Supplementary material for: AKT Signaling Differentially Regulates the Expression of Two Evolutionarily Conserved Wnt5a Isoforms in Lung Mesenchymal Cells
Source: Cells. 2026 May 4;15(9):843. doi: 10.3390/cells15090843 (PMC13162999; doi:10.3390/cells15090843)
Supplement: Supplementary file 1 [file cells-15-00843-s001.zip › cells-4246552-supplementary.pdf]

Supplementary Materials:

Gene name (mouse)	qRT-PCR Primer Sequences
TBP	Forward: 5’-CCAATGACTCCTATGACCCCTA-3’<br>Reverse: 5’-CAGCCAAGATTCACGGTAGAT-3’
Acta2 (aSMA)	Forward: 5’- CCCACCCAGAGTGGAGAA-3'<br>Reverse: 5’- ACATAGCTGGAGCAGCGTCT-3'
Mki67	Forward: 5’-GGCGTTATCCCAGGAGACT-3’<br>Reverse: 5’-GCTGTCCTCAAGACAATCATC-3’
Pai1	Forward: 5’-GGGACGAAACTGGAGATGTTAT-3’<br>Reverse: 5’-GGGATGCTGGTTGGAAAGA-3’
Col1a1	Forward: 5’-CATGTTCAGCTTTGTGGACCT-3’<br>Reverse: 5’-GCAGCTGACTTCAGGGATG-3’
Tgfbi	Forward: 5’-GCTGCGAGTCTTTGTTTATCG-3’<br>Reverse: 5’-CCTCTTATCATGGGCAGCA-3’
IL-1b	Forward: 5’-ACGGACCCCAAAAGATGAAG-3’<br>Reverse: 5’-TTCTCCACAGCCACAATGAG-3’
IL-6	Forward: 5’-TTGCCTTCTTGGGACTGATG-3’<br>Reverse: 5’-AGGTCTGTTGGGAGTGGTAT-3’
Tcf7	Forward: 5’-GCGGGATAACTACGGAAAGAAG-3’<br>Reverse: 5’-GAAGGAGGGCAACAGAAGATAC-3’
Wnt5a (total)	Forward: 5’-ATGAAGCAGGCCGTAGGAC-3’<br>Reverse: 5’-CTTCTCCTTGAGGGCATCG-3’
Wnt5a-L	Forward: 5’-GTGGCGACTTCCTCTCCGT-3’<br>Reverse: 5’-CGGTCCCCAAAGCCACT-3’<br>Probe: 5’-CCCCTCGCCATGAAGAAGCCCA-3’
Wnt5a-S	Forward: 5’-ACTTGTTGCTCCGGCCC- 3’<br>Reverse: 5’-CGGTCCCCAAAGCCACT-3’<br>Probe: 5’-AGAAGCCCATTGGAATATTAAGCCCGG-3’

Supplemental Table S1: Sequences of qRT-PCR primers

**Anitbody & Reagents**	**Company**	**Catelog #**
Mouse anti-ACTA2	MilliporeSigma	A5228
Rabbit anti-P-ERK	Cell Signaling Technology	4377
Rabbit anti-P-AKT	Cell Signaling Technology	3787
Rabbit anti-beta-actin	Abcam	AB8227
Mouse anti-GAPDH	MilliporeSigma	MAB374
Mouse-anti-GFP	Santa Cruz	SC9996
Goat-anti-TAGLN	Abcam	ab10135
Rabbit-anti-AQP5	Alomono Labs	AQP-005
Goat-anti-EMCN	R&D Systems	AF4666
U0126	MilliporeSigma	19-147
SIS3	MilliporeSigma	566405
PF04691502	MilliporeSigma	PZ0235
Imatinib	Selleckchem	S1067
PDGF-AA	ThermoFisher Scientific	100-13A
TGF-beta1 (human)	R & D systems	240-B

Supplemental Table S2: Antibodies & Reagents
